# Ipilimumab induced digital vasculitis

**DOI:** 10.1186/s40425-018-0321-2

**Published:** 2018-02-12

**Authors:** Amrita Padda, Elena Schiopu, Justin Sovich, Vincent Ma, Ajjai Alva, Leslie Fecher

**Affiliations:** 1 Division of Rheumatology, Department of Internal Medicine, University of Michigan, Floor 3, Reception A, 1500 E Medical Center Drive, SPC 5342, Ann Arbor, MI 48109 USA; 2 Department of Internal Medicine, University of Michigan, Ann Arbor, MI USA; 3 Division of Hematology Oncology, Department of Internal Medicine, University of Michigan, Ann Arbor, MI USA

**Keywords:** Ipilimumab, Immune related adverse events (IRAEs), Vasculitis

## Abstract

**Background:**

Immune check point inhibitors (ICIs) have emerged as a new therapeutic paradigm for a variety of malignancies including metastatic melanoma. As the use of ICIs expand, immune-mediated adverse events are becoming a common occurrence.

**Case presentation:**

We describe the first reported patient with small vessel vasculitis, manifested by digital ischemia, following treatment with high dose Ipilimumab for resected stage IIIB/C melanoma. This patient received high dose steroids, five-day intravenous (IV) Epoprostenol protocol, botulinum toxin injections, and Rituximab 375 mg/m^2^ weekly for four cycles. With this treatment regimen, the digital ischemia did not progress proximally, but she did require multiple distal digit amputations about six months after the onset of her symptoms.

**Conclusions:**

Prompt identification and management of immune related adverse events (IRAEs) are critical to optimal patient management. This patient’s vasculitis did not reverse, but was likely halted and stabilized with multiple immunosuppressive medications.

## Background

Ipilimumab (Yervoy®) is approved by the Food and Drug Administration (FDA) for the treatment of resected stage III melanoma and advanced unresectable melanoma. It is a fully human monoclonal antagonistic antibody which targets cytotoxic T lymphocyte antigen 4 (CTLA-4) on T cells and blocks the CTLA-4 interaction with its ligand CD80. CTLA-4 is an immune check point molecule which downregulates pathways of T cell activation. Therefore, when CTLA-4 is blocked with Ipilimumab, the T lymphocyte inhibitory pathway is hindered, and the immune response is enhanced, allowing T lymphocytes to destroy cancer cells [1]. Melanoma incidence continues to rise and metastatic melanoma results in approximately 53,000 deaths per year worldwide as estimated by the World Health Organization [2]. Ipilimumab was the first therapeutic agent to demonstrate an overall survival benefit in the treatment of advanced, unresectable melanoma [3]. It is currently approved by the FDA at a dose of 3 mg/kg in the metastatic setting. More recently, Ipilimumab 10 mg/kg demonstrated an improved median relapse free survival of 26.1 months compared to 17.1 months for placebo in resected stage III cutaneous melanoma in the European Organization for Research and Treatment of Cancer (EORTC)18,071; this study led to its approval by the FDA for this indication [4]. An update for this study was recently published and reported a five year relapse free survival of 40.8% in the Ipilimumab group compared to 30.3% in the placebo group, with a median follow up of 5.3 years [5]. Five-year overall survival in the Ipilimumab group was 65.4% versus 54.4% in the placebo group. No vascular toxicities of any grade were reported. Please see Table 1 for adverse events.

**Table 1:** Grade 3/4/5 toxicities from the E1609 trial and EORTC trial. These studies are in the setting of resected patients (adjuvant)

	E1609 trial^a^ (Safety Data *n* = 1019) (Total Enrollment = 1673)		EORTC 18071 trial (*n* = 945)	
Treatment type	Ipi 3 mg/kg	Ipi 10 mg/kg	Ipi 10 mg/kg	Placebo
Number of patients	516	503	471	474
Adverse event of any grade	98.4%	100%	(465 99%)	432 (91%)
Treatment-related AE (any grade)	96%	98.8%		
Grade 3 adverse events^b^	37%	57%		
Grade 4 adverse events^b^				
Immune related adverse events (grade 3/4)	18.8%	34%	196 (41.6%)	13 (2.7%)
Gastrointestinal adverse event^b^	12.0%	18.5%	76 (16%)	4 (< 1%)
Hepatic adverse events^b^	3.1%	7.8%	51 (11%)	1 (< 1%)
Endocrine adverse events^b^	6.6%	12.4%	37 (8%)	1(< 1%)
Neurologic adverse events^b^	2.0%	1.6%	9 (1.9%)	0 (0%)
Treatment related Adverse event leading to discontinuation of treatment	35%	54%	240 (51%)	22(4.6%)
Death due to treatment related adverse events	2 (0.4%)	8 (1.6%)	5 (1.1%)	0

^a^Abstract available only for the E1609 trial

^b^Grade 3/4 adverse events

Preliminary safety data from an unplanned interim analysis for Ipilimumab-treated subjects was recently presented from the 1609 trial sponsored by the Eastern Cooperative Oncology Group at the American Society for Clinical Oncology [6]. This phase III study in subjects with resected stage III and IV melanoma randomized 1673 patients to high dose interferon (HDI), Ipilimumab 3 mg/kg, or Ipilimumab 10 mg/kg, with co-primary endpoints of relapse free survival and overall survival. They reported safety data for 1019 subjects treated at either dose of Ipilimumab, as well as relapse free survival data for 773 concurrently randomized subjects with a median follow up of 3.1 years. There were two deaths (0.4%) in the lower dose Ipilimumab arm due to colitis and eight (1.6%) in the higher dose Ipilimumab arm: five subjects with colitis, one pneumonitis, one thromboembolic event with hypophysitis, and one cardiac event. This unplanned exploratory analysis showed no difference in relapse free survival between the low dose and high dose Ipilimumab, however additional follow up is needed. Of note, the Ipilimumab 10 mg/kg arm accrual was suspended for approximately 2 months due to toxicity (Table 1). These adjuvant toxicity rates are at least equivalent and possibly greater than those reported in the advanced, unresectable melanoma setting with grade 3/4 rates reported at 27-58% for 10 mg/kg [7–10].

## Case presentation

We report a case of a 52-year-old Caucasian woman who was diagnosed with stage IIIB/C melanoma from a regressed primary of the abdomen when she presented with a bulky left groin mass. Imaging demonstrated a 6.5 cm lobulated mass in the left groin involving soft tissue and probable lymph nodes. Excisional biopsy revealed two lymph nodes with metastatic melanoma; tumor cells were positive for Melan-A and SOX-10, and negative for CD45, Cytokeratin AE1/AE3. The patient reported a long standing black skin lesion at the lower abdomen that had previously grown and bled, but more recently had started to fade. This lesion was biopsied and demonstrated superficial dermal fibrosis with tumoral melanosis and pigment laden macrophages (melanophages), consistent with a regressed melanocytic lesion. She had no distant metastases on staging and underwent a wide local excision of the regressed primary site and radical resection of the inguinal mass with complete lymph node dissection, followed by reconstruction of the left groin with a sartorius flap. Pathology of the 8.9 cm subcutaneous mass revealed a spindle cell (sarcomatoid) melanoma involving lymph node tissue, possible aggregate of matted lymph nodes with extranodal extension, and multiple additional lymph nodes without melanoma. She had no known history of autoimmune conditions or prior Raynaud’s phenomenon.

The first high dose Ipilimumab of 10 mg/kg was administered at an outside hospital followed by limited loose bowel movements and mouth soreness. One week after the second Ipilimumab infusion (at 3-week intervals), she developed myalgias, arthralgias, rash, vision changes, jaw pain, and discoloration of several upper and lower limb digits. Initially, the digital symptoms included intermittent red and blue color changes, worse in the cold, and better with warmth. Two weeks after the second infusion, her oncologist initiated Amlodipine 10 mg daily, Aspirin 81 mg daily, and Prednisone 10 mg daily for suspected immune-mediated Raynaud’s phenomenon. Serologic workup at the initial presentation included a negative antinuclear antibody, rheumatoid factor, anti-cyclic citrullinated peptide antibody, cytoplasmic and perinuclear anti-neutrophil cytoplasmic antibodies, cryoglobulins, and hepatitis C antibody and ribonucleic acid (RNA); serum and urine protein electrophoresis were within normal limits. She reported immediate improvement of the myalgias, rash, arthralgias and jaw pain, but she had progression of digital pain and discoloration, therefore her oncologist administered 500 mg of IV Methylprednisolone and increased her daily Prednisone to 60 mg for a presumed Ipilimumab immune related adverse event (IRAE). Her digits did not improve, and she received a second 500 mg dose of IV Methylprednisolone three days later. The lower extremity digital pain resolved, but the upper extremity digital pain became so severe that she required high doses of morphine.

**Fig. 1:**
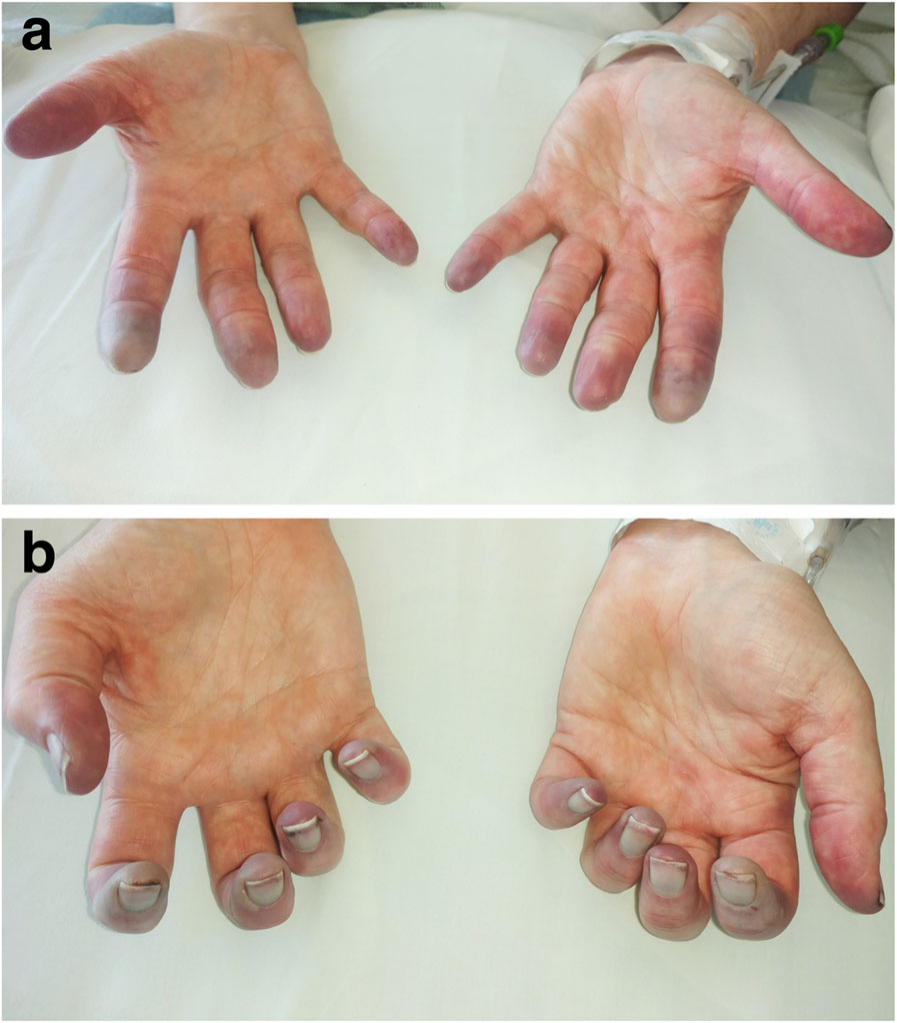
(Panel **a** and **b**). This picture was taken four weeks after her second Ipilimumab infusion (week 26 on timeline). Physical exam reveals acrocyanosis of all digits with small ulcers of the right second and fourth fingertips

**Fig. 2:**
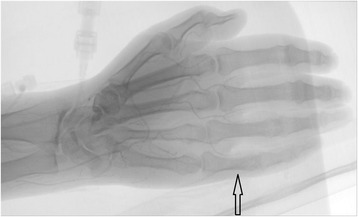
Conventional angiogram of the left arm was performed five weeks after her second Ipilimumab infusion (end of week 26 on timeline). There is severely diminished flow in the digital arteries of the left hand beyond the level of all proximal interphalangeal joints (black arrow), consistent with small vessel occlusive disease

She was admitted for inpatient management two days after her outpatient steroid infusions. Physical exam revealed acrocyanosis of all upper extremity digits (Fig. 1a & 1b). She was initiated on 2 mg/kg of IV Methylprednisolone and calcium channel blockade. Lower extremity digits were less affected and demonstrated the greatest improvement. The upper extremity digital pain and acrocyanosis persisted. She was treated with one dose of 1000 mg of IV Methylprednisolone with limited benefit, and thus continued daily 2 mg/kg dosing. Arterial duplex dopplers of upper and lower extremities were normal. Transthoracic echocardiogram was negative for thrombus or mass. Conventional angiogram was performed on the left upper extremity; it revealed severely diminished blood flow in the digital arteries beyond the proximal interphalangeal joints, and the Verapamil challenge did not increase blood flow (Fig. 2). Findings on the conventional angiogram were consistent with small vessel occlusive disease. Extensive autoantibody panels (inclusive of myeloperoxidase, proteinase 3, antinuclear, anti-centromere, anti-topoisomerase I, Beta-2-glycoprotein 1, anti-cardiolipin, anti-neutrophil cytoplasmic) and viral panels (human immunodeficiency virus, hepatitis panels) were negative. A full coagulopathy work-up was unrevealing. Given concern for ongoing immune-mediated vasculitis, the patient also received a 5-day course, 6-h per day, of Epoprostenol at a rate of 3 ng/kg/min. Fifty units of botulinum toxin A in 10 mL of normal saline was injected into each hand via palmar approach focusing on the proximal aspect of each digital artery. After six days of IV steroids, she was transitioned to oral Prednisone 100 mg (1 mg/kg) daily. She received four cycles of Rituximab at 375 mg/m^2^, at approximately one-week intervals. Her Prednisone was tapered to 10 mg daily over the course of seven weeks. She then developed an IRAE of pneumonitis, which quickly improved when the Prednisone was increased to 50 mg daily. During steroid treatment, she was also on Alendronate and calcium for osteoporosis prophylaxis, Bactrim for pneumocystis pneumonia prophylaxis, and Omeprazole for gastrointestinal protection.

**Fig. 3:**
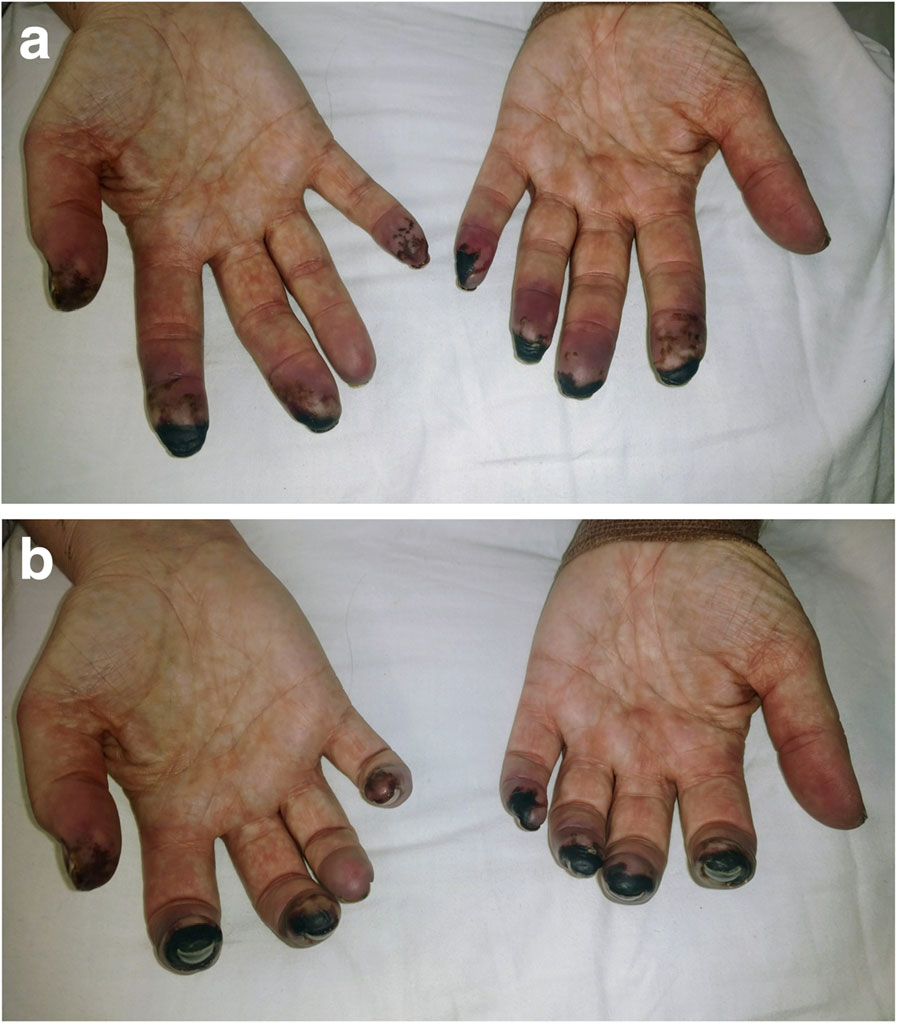
(Panel **a** and **b**) This picture was taken nine weeks after her second Ipilimumab infusion (week 31 on timeline). The patient is status-post high dose steroids and four cycles of Rituximab. The exam appeared worse with dry gangrene of the fingertips, secondary to the natural evolution of skin changes with distal digital ischemia. We believe that the vasculitic process was halted, as she did not develop further proximal digital ischemia

After the four cycles of Rituximab, we believe that the vasculitic process and additional damage were halted, as she did not develop further proximal digital ischemia or other systemic symptoms. The exam appeared worse with dry gangrene of the fingertips, consistent with the natural evolution of skin changes with distal digital ischemia, as shown in Fig. 3a & b (Taken 5 weeks after Fig. 1), likely reflecting consequences of the initial injury. She did ultimately require distal digit amputations. See Table 2 for the chronological outline of the case presentation.

**Table 2:** Summarized timeline of case presentation

Week 0	Presented with left groin mass. Excisional lymph node biopsy was consistent with metastatic melanoma.
Week 4	Radical resection of melanoma with wide local excision of regressed primary and complete lymph node dissection.
Week 19	First cycle of Ipilimumab 10 mg/kg. Side effects included mild diarrhea and mouth soreness.
Week 22	Second cycle of Ipilimumab 10 mg/kg.
Week 23	Symptoms of myalgias, arthralgias, rash, vision changes, jaw pain, and discoloration of several upper and lower limb digits.
Week 24	Amlodipine 10 mg daily, Aspirin 81 mg daily, and Prednisone 10 mg daily initiated for suspected Raynaud’s phenomenon. Digital pain and discoloration progressed.
Week 25	She received Methylprednisolone 500 mg IV followed by oral Prednisone 60 mg daily. Additional dose of 500 mg Methylprednisolone IV given later in the week. Lower extremity digital pain resolved, upper extremity digital pain progressed.
Week 26	Admitted. Initiated on Methylprednisolone 2 mg/kg/day IV, calcium channel blockade, and nitropaste. Administered an additional Methylprednisolone 1000 mg dose. Epoprostenol initiated for a 5-day course. Botulinum toxin A was injected into each hand. Refer to Fig. 1 for the physical exam and Fig. 2 for the conventional angiogram.
Week 27	Transitioned to oral Prednisone 100 mg (1 mg/kg) daily and Sildenafil 20 mg BID.
Week 27-30	Initiated on weekly Rituximab 375 mg/m2. Refer to Fig. 3 for the physical exam at week 31.
Week 32	Prednisone was tapered down to 10 mg daily.
Week 34	Developed IRAE of pneumonitis, prednisone was increased to 50 mg daily and symptoms improved.
Week 48-52	Surgical amputation of multiple distal digits.
Week 49	Prednisone completely weaned off.

## Discussion and conclusions

Immune related adverse events (IRAEs) are common with checkpoint inhibitors, and can range from mild to severe and life threatening [11, 12]. While there are common patterns in presentation, it is not possible to predict specific toxicities at this time. Skin, gastrointestinal tract, liver and endocrine glands are commonly affected. In general, IRAEs are treated with steroids which can require high doses with slow tapers at least over one month. Some IRAEs can be refractory and require additional immunosuppressive or immune-modulating agents including Infliximab or Mycophenolate [11–13]. Immune-related toxicities have been reported involving nearly all organ systems, including rare cardiac and neurologic toxicities [14]. Inflammatory arthritis mimicking rheumatoid arthritis and sicca syndrome have frequently been described in Ipilimumab use [15]. However, IRAEs involving blood vessels, such as vasculitides are quite rare and reported at a less than 1% incidence [16]. The most common vascular IRAE reported to date is giant cell arteritis/temporal arteritis [17]. A recent review addresses the molecular mechanism of how immune checkpoint inhibitors increase anti-vascular immunity risk, specifically in causing medium and large cell vasculitis, like giant cell arteritis [18]. In a search across PubMed, there have only been three case reports of vasculitis in Ipilimumab use. One case was isolated lymphocytic uterine vasculitis [19]. The other two cases were polymyalgia rheumatica with giant cell arteritis, both patients highly responsive to prednisone [20].

To our knowledge, this is the first reported case of an Ipilimumab IRAE causing small vessel vasculitis manifesting as digital ischemia. This case illustrates the current debate in melanoma oncology regarding the risk-benefit profile of adjuvant high dose Ipilimumab. Multiple studies of programmed death receptor-1 inhibitors in the adjuvant setting are completed with preliminary data or ongoing with pending data and further data from ECOG 1609 trial is forthcoming. This case highlights the importance of close monitoring of patients on immune checkpoint inhibitor therapy and prompt diagnosis and management of immune related adverse events.

## Abbreviations

CTLA-4: Cytotoxic T-lymphocyte antigen 4
ICI: Immune checkpoint inhibitor
IRAE: Immune related adverse event
IV: Intravenous
